# Supplementary material for: Dynamic transcriptomic profiles of zebrafish gills in response to zinc supplementation
Source: BMC Genomics. 2010 Oct 11;11:553. doi: 10.1186/1471-2164-11-553 (PMC3091702; doi:10.1186/1471-2164-11-553)
Supplement: Additional file 2 — Interactive Direct Interaction Network representing the molecular interactions between zinc, copper, iron, calcium and proteins encoded by transcripts changed by zinc supplementation. Mini web-site containing index.html and hyperlinked pages in subdirectory describing a Direct Interaction Network automatically generated based on curated interactions contained within the proprietary PathwayArchitect database. Ovals represent proteins and the circles symbolize metal ions. Objects are coloured by their abundance in zebrafish at the time-point they were significantly different from the control is a scale from -4 fold (dark green) to +4 fold (dark red). Where significant differences were found at more than one time-point, the colour overlay shows expression at the first instance. Dark blue squares denote 'binding', and light blue squares 'expression'; green squares stand for 'regulation', green diamonds for 'metabolism', and green circles for 'promoter binding'. Arrow heads indicate directionality of the interaction where annotated. All nodes and edges can be further interrogated by selecting the relative area of the image. [file 1471-2164-11-553-S2.zip › PathwayArchitect Zn xs DIN/89657.html]

# PROTEIN: CUGBP2

|  |  |
| --- | --- |
| Name | CUGBP2 |
| Type | PROTEIN |
| Description | CUG triplet repeat, RNA binding protein 2 |
| Note | Members of the CELF/BRUNOL protein family contain two N-terminal RNA recognition motif (RRM) domains, one C-terminal RRM domain, and a divergent segment of 160-230 aa between the second and third RRM domains. Members of this protein family regulate pre-mRNA alternative splicing and may also be involved in mRNA editing, and translation. Alternative splicing results in multiple transcript variants encoding different isoforms. |
| Alias | elav-type RNA-binding protein 3 |
|  | BRUNOL3 |
|  | CUG triplet repeat,RNA binding protein 2 |
|  | B230345P09Rik |
|  | ELAV-type RNA-binding protein 3 |
|  | Napor |
|  | neuroblastoma apoptosis-related RNA-binding protein isoform 4 |
|  | CUG triplet repeat,RNA-binding protein 2 |
|  | ETR-3 |
|  | Etr3 |
|  | CUG triplet repeat RNA-binding protein 2 |
|  | CUG triplet repeat, RNA-binding protein 2 |
|  | NAPOR |
|  | neuroblastoma apoptosis-related RNA-binding protein |
|  | CUG triplet repeatRNA-binding protein 2 |
|  | C88023 |
|  | Napor-2 |


---

|  |  |
| --- | --- |
| GO Component | soluble fraction |
|  | nucleus |


---

|  |  |
| --- | --- |
| GO ID | GO:0006397 |
|  | GO:0007528 |
|  | GO:0006376 |
|  | GO:0005634 |
|  | GO:0003723 |
|  | GO:0000398 |
|  | GO:0008016 |
|  | GO:0005625 |


---

|  |  |
| --- | --- |
| MIM | MIM:602538 |


---

|  |  |
| --- | --- |
| Connectivity | 31 |


---

|  |  |
| --- | --- |
| Entrez ID | 14007 |
|  | 10659 |
|  | 29428 |


---

|  |  |
| --- | --- |
| Agilent ID | A\_14\_P201859 |
|  | A\_14\_P108315 |
|  | A\_14\_P110137 |
|  | A\_14\_P102379 |
|  | A\_53\_P164582 |
|  | A\_44\_P456728 |
|  | A\_51\_P182471 |
|  | A\_23\_P115645 |
|  | A\_14\_P100142 |
|  | A\_53\_P150258 |
|  | A\_52\_P478051 |
|  | A\_43\_P11855 |
|  | A\_23\_P202071 |
|  | A\_14\_P121122 |
|  | A\_53\_P103267 |
|  | A\_14\_P138358 |
|  | A\_14\_P129768 |
|  | A\_52\_P499551 |
|  | A\_52\_P100909 |
|  | A\_14\_P102080 |
|  | A\_53\_P159015 |


---

|  |  |
| --- | --- |
| Cellular Localization | Nucleus |
|  | Organelle |
|  | Cell |


---

|  |  |
| --- | --- |
| Pathway | Zn xs inventory |
|  | Zn xs DIN |


---

|  |  |
| --- | --- |
| GO Process | neuromuscular junction development |
|  | nuclear mRNA splicing, via spliceosome |
|  | mRNA processing |
|  | regulation of heart contraction rate |
|  | mRNA splice site selection |


---

|  |  |
| --- | --- |
| UniGene | Rn.30004 |
|  | Hs.309288 |
|  | Mm.147091 |


---

|  |  |
| --- | --- |
| Affymetrix Probeset ID | 110034\_at |
|  | 114681\_at |
|  | 1375591\_at |
|  | 1380670\_at |
|  | 1387879\_a\_at |
|  | 1387880\_at |
|  | 1388195\_at |
|  | 1389867\_at |
|  | 1396654\_at |
|  | 1397217\_at |
|  | 1397673\_at |
|  | 1423895\_a\_at |
|  | 1440322\_at |
|  | 1441072\_at |
|  | 1443545\_at |
|  | 1444152\_at |
|  | 1444533\_at |
|  | 1445306\_at |
|  | 1446670\_at |
|  | 1447531\_x\_at |
|  | 1450069\_a\_at |
|  | 1451154\_a\_at |
|  | 1451155\_at |
|  | 1560049\_at |
|  | 1457553\_at |
|  | 1458263\_at |
|  | 1554569\_a\_at |
|  | 1556323\_at |
|  | 1565599\_at |
|  | 166401\_r\_at |
|  | 202156\_s\_at |
|  | 202157\_s\_at |
|  | 202158\_3p\_s\_at |
|  | 202158\_s\_at |
|  | 214405\_at |
|  | 215144\_at |
|  | 215147\_at |
|  | 227178\_at |
|  | 231109\_at |
|  | 234151\_at |
|  | 240665\_at |
|  | 242268\_at |
|  | 32851\_at |
|  | 34532\_at |
|  | 35087\_at |
|  | 44375\_at |
|  | 45208\_at |
|  | 52674\_at |
|  | 55590\_at |
|  | 68367\_at |
|  | 70284\_at |
|  | 73742\_at |
|  | 90262\_at |
|  | 92007\_at |
|  | 93892\_at |
|  | 97255\_at |
|  | AJ010351\_s\_at |
|  | AJ010386\_s\_at |
|  | g1568642\_3p\_a\_at |
|  | g5729815\_3p\_a\_at |
|  | g5729815\_3p\_s\_at |
|  | Hs.113166.0.S1\_3p\_at |
|  | Hs.12533.0.S1\_3p\_at |
|  | Hs2.113166.2.A1\_3p\_at |
|  | Hs.211610.0.A2\_3p\_a\_at |
|  | Hs.21248.0.A1\_3p\_at |
|  | Hs2.211610.4.S1\_3p\_s\_at |
|  | Hs2.384584.1.A1\_3p\_at |
|  | Hs2.384584.1.A1\_3p\_s\_at |
|  | Hs.277419.0.S1\_3p\_at |
|  | Hs.306699.0.S1\_3p\_at |
|  | Hs.4268.0.A1\_3p\_at |
|  | Hs.90043.0.A1\_3p\_at |
|  | rc\_AA900699\_at |
|  | rc\_AA956703\_at |
|  | rc\_AA956703\_g\_at |
|  | rc\_AI101245\_at |
|  | rc\_AI178706\_at |
|  | U69546\_at |
|  | 1396855\_at |
|  | 1557422\_at |
|  | 72887\_at |
|  | rc\_AI058715\_at |
|  | 66305\_at |
|  | Hs2.117474.1.A1\_3p\_at |
|  | Hs2.308017.1.S1\_3p\_at |
|  | RC\_AA233774\_at |
|  | RC\_AA279463\_at |
|  | RC\_AA403114\_at |
|  | RC\_F02584\_at |
|  | RC\_N78694\_at |
|  | RC\_R56068\_at |
|  | RC\_R59360\_s\_at |
|  | RC\_T84491\_at |
|  | TC21050\_at |
|  | TC31667\_at |
|  | TC31668\_at |


---

|  |  |
| --- | --- |
| GO Function | RNA binding |


---

|  |  |
| --- | --- |
| Nucleotide | AK164914 |
|  | AF090696 |
|  | U69546 |
|  | NM\_017197 |
|  | AK151818 |
|  | CR936676 |
|  | AK162651 |
|  | AK161349 |
|  | AF432906 |
|  | AK209314 |
|  | AL157704 |
|  | AF090695 |
|  | AF169013 |
|  | NM\_010160 |
|  | AK046163 |
|  | AK037436 |
|  | AJ010386 |
|  | BC026856 |
|  | AK153267 |
|  | AK164112 |
|  | AF295068 |
|  | AJ010351 |
|  | AK152628 |
|  | NM\_001025076 |
|  | AK138189 |
|  | AK197068 |
|  | AK207977 |
|  | Y18298 |
|  | AL136320 |
|  | AF036956 |
|  | AK137292 |
|  | NM\_006561 |
|  | AK038251 |
|  | AF090693 |
|  | BC036391 |
|  | AK160861 |
|  | AF090694 |
|  | NM\_001025077 |
|  | AK159996 |
|  | AK135166 |
|  | AK134815 |
|  | AK172648 |
|  | AK193426 |
|  | AK139126 |
|  | AF090697 |


---

|  |  |
| --- | --- |
| Protein | BAE30715 |
|  | NP\_034290 |
|  | NP\_058893 |
|  | CAA09102 |
|  | AAD02074 |
|  | AAH36391 |
|  | AAK92699 |
|  | CAI20168 |
|  | AAK72223 |
|  | CAI20169 |
|  | AAD13764 |
|  | AAB09040 |
|  | CAA09103 |
|  | BAE37963 |
|  | CAA77110 |
|  | AAD13762 |
|  | BAE31371 |
|  | AAD13760 |
|  | BAE31857 |
|  | NP\_001020247 |
|  | AAH26856 |
|  | NP\_001020248 |
|  | BAE23295 |
|  | AAD13761 |
|  | AAK72224 |
|  | CAH70230 |
|  | BAE36341 |
|  | AAD13763 |
|  | BAE36053 |
|  | AAL27627 |
|  | NP\_006552 |
|  | AAF89096 |
|  | CAH70231 |


---

|  |  |
| --- | --- |
| Organism | Mammal |


---

|  |  |
| --- | --- |
| Location | chromosome 17, 17q12.3 (Rattus norvegicus) |
|  | chromosome 2, 2 A2-A3 (Mus musculus) |
|  | chromosome 10, 10p13 (Homo sapiens) |


---

|  |  |
| --- | --- |
